# ATR Inhibition Broadly Sensitizes Soft-Tissue Sarcoma Cells to Chemotherapy Independent of Alternative Lengthening Telomere (ALT) Status

**DOI:** 10.1038/s41598-020-63294-z

**Published:** 2020-05-04

**Authors:** Audrey Laroche-Clary, Vanessa Chaire, Stéphanie Verbeke, Marie-Paule Algéo, Andrei Malykh, François Le Loarer, Antoine Italiano

**Affiliations:** 1 INSERM ACTION U1218, Institut Bergonié, Bordeaux, France; 2 Sarcoma Unit, Institut Bergonié, Bordeaux, France; 3 University of Bordeaux, Bordeaux, France; 4 Capital Biosciences Rockville, Maryland, USA

**Keywords:** Cancer therapy, Sarcoma

## Abstract

Only few drugs have shown activity in patients with advanced soft-tissue and the median overall survival is only 18 months. Alterations of genes involved in the DNA damage repair pathway have been associated with sarcoma risk and prognosis. ATR plays a crucial role in maintaining genomic integrity by responding to a large spectrum of DNA damage, including double strand breaks (DSBs) that interfere with replication. The objective of this study is to evaluate the pre-clinical activity of ATR inhibition in soft tissue sarcomas (STS). We explored the ability of the ATR inhibitor, VE-822, to prevent chemotherapy-induced intra-S-phase checkpoint activation and evaluated the antitumor potential of this combination *in vitro* and *in vivo* in STS cell lines and in a patient-derived xenograft model. The combination of VE-822 and gemcitabine *in vitro* was synergistic, inhibited cell proliferation, induced apoptosis, and accumulated in the S phase of the cell cycle with higher efficacy than either single agent alone. The combination also resulted in enhanced γH2AX intranuclear accumulation as a result of DNA damage induction. These effects were unrelated to the alternative lengthening of telomeres pathway. *In vivo*, the combination of VE-822 and gemcitabine significantly enhanced tumor growth inhibition and progression-free survival in an aggressive model of undifferentiated pleomorphic sarcoma. The combination of ATR inhibitor and chemotherapy is beneficial in pre-clinical models of soft-tissue sarcoma and deserves further exploration in the clinical setting.

## Introduction

Surgical resection represents the cornerstone of treatment of patients with localized soft-tissue sarcoma (STS). However, up to 40% of patients who underwent optimal surgery will develop metastatic disease^1^. Only few drugs including doxorubicin, or gemcitabine have shown activity in the advanced setting^2,3^ and the median overall survival has only slightly improved in the last 20 years from 12 months to 18 months^2^. There is a crucial need for new and effective drugs for patients with advanced STS.

Gene expression profiling of a large cohort of STS allowed the identification and validation of a 67 gene signature of chromosome instability named CINSARC (for genome Complexity INdex in SARComas) and which is the most significant predictor of metastasis free survival in these tumors^4^. Many of the genes identified encode for proteins involved in DNA repair.

Moreover, a recent international study has shown that germline variants in several genes encoding proteins involved in DNA repair such as *BRCA2*, *ATM*, *ATR*, and *ERCC2*, contributed significantly to sarcoma risk^5^.

ATR plays a crucial role in maintaining genomic integrity by responding to a large spectrum of DNA damage, including double strand breaks (DSBs) that interfere with replication. There are no specific data related to the impact of ATR inhibition in a panel of STS pre-clinical models. Here, we used the ATR inhibitor, VE-822, to investigate the effect of ATR inhibition in STS cell lines and patient derived- xenograft. We also explored the ability of VE-822 to sensitize STS cells to chemotherapy, and characterized the features of the synergy to provide a rational for clinical studies.

## Results

### VE-822 has antiproliferative activity and induces apoptosis and S-phase cell cycle delay in STS cells

We studied the sensitivity of 8 STS cell lines to VE-822 and gemcitabine. The IC50 values for VE-822 ranged between 1.3 and 12 µM (Table 1). All the cell lines had impaired TP53 pathway has a result of *TP53* mutation/deletion or *MDM2* amplification except for two cell lines (IB114 and IB128).

**Table 1:** Activity of VE-822 and gemcitabine in soft-tissue sarcoma cells.

Cell line ID	Histological subtype	*TP53* mutational status	*MDM2* amplification status	IC50 VE-822 (µM)	IC50 Gemcitabine (nM)	Combination index	ALT * status
IB111	Dedifferentiated liposarcoma	Wild-type	Amplified	4.7	2.8	0.49	positive
IB112	Leiomyosarcoma	Null	Normal	5.8	1.1	0.67	positive
IB114	Myxofibrosarcoma	Wild-type	Gain	1.3	5.4	0.8	negative
IB115	Dedifferentiated liposarcoma	Wild-type	Amplified	1.5	1.9	0.71	positive
IB128	Extra-skeletal osteosarcoma	Wild-type	Normal	3.1	3.3	0.45	negative
IB134	Leiomyosarcoma	S215R	Gain	12	3.7	0.96	positive
IB136	Leiomyosarcoma	Null	Gain	5.1	8.2	0.5	negative
93T449	Well differentiated liposarcoma	Wild-type	Amplified	8	3.7	0.91	negative

*ALT: alternative lengthening telomere.

**Figure 1:**
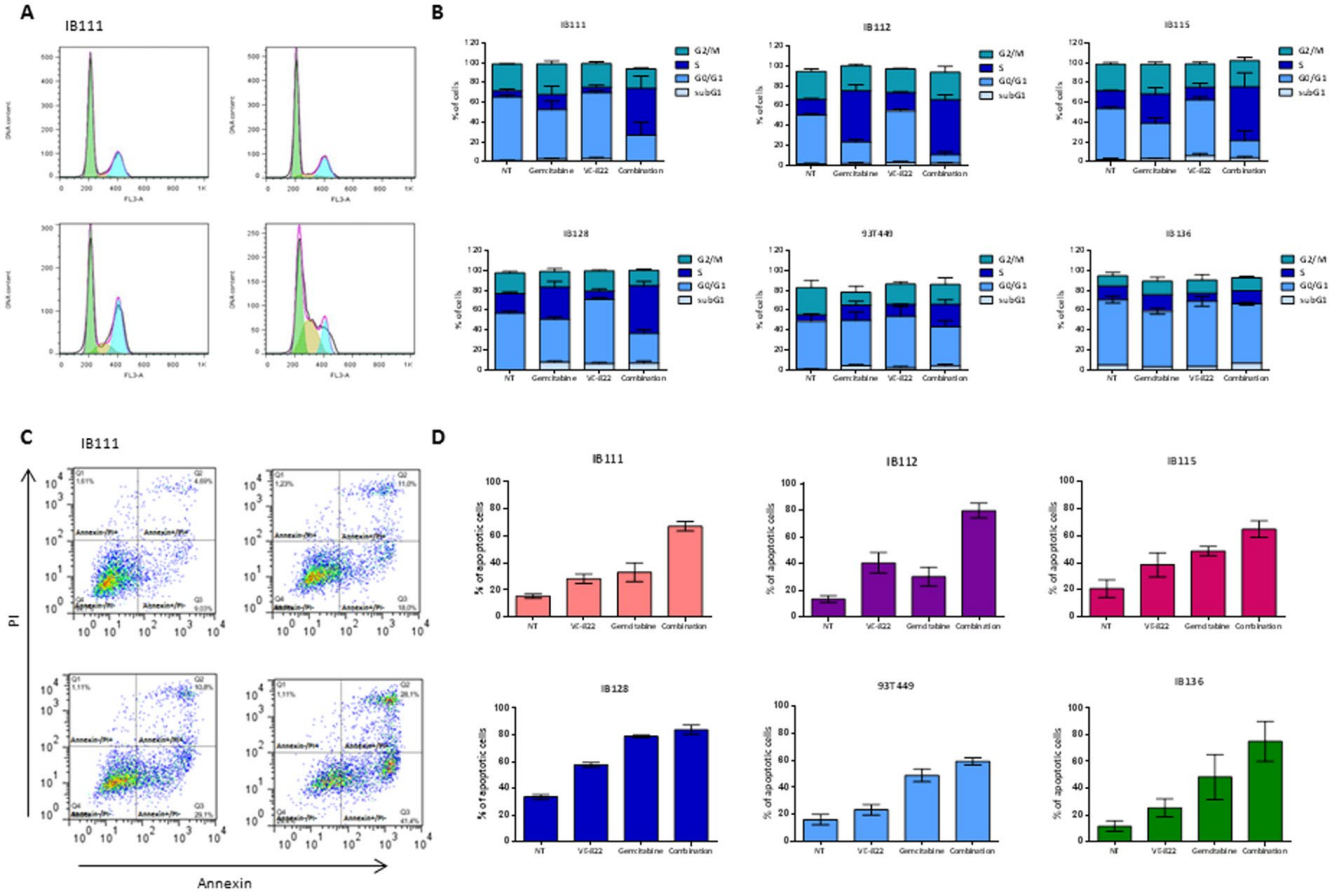
Effect of VE-822 as a single agent and in combination with gemcitabine on cell cycle and apoptosis in soft-tissue sarcoma cells. **(A)** Cell-cycle profile after 48 h of treatment with gemcitabine and/or VE-822 analyzed by PI incorporation and flow cytometry in the IB111 cell line. **(B)** Effect of gemcitabine and VE-822 combination on cell cycle progression in 6 STS cell lines: IB111, IB112, IB115, IB128, 93T449 and IB136. Cell-cycle distribution was calculated from the flow cytogram. **(C)** Annexin V FITC-A vs propidium iodide-A plots from the gated cells show the populations corresponding to viable and non-apoptotic (Annexin V−PI−), early (Annexin V + PI−), and late (Annexin V + PI+) apoptotic cells in the IB111 cell line. **(D)** Quantification of apoptotic cells after 72 h of treatment with VE-822 or Gemcitabine alone or a combination of the two drugs.

We next assessed the impact of ATR inhibition on cell cycle progression. In response to VE-822, cells accumulated in G0/G1 consistent with G1/S cell cycle arrest. By using the Annexin V-based detection assay, apoptosis was also detected under these conditions (Fig. 1C,D).

### Sensitivity of STS cells to VE-822 is not related to alternative lengthening of telomeres

Given that the cell death induced by ATR inhibitors was suggested to be highly selective for cancer cells that rely on alternative lengthening telomere (ALT)^6^, we assessed the ALT status of our panel of cell lines by using the C-Circle assay as previously described^7^. We then explored the correlation between VE-822 sensitivity and ALT status. The VE-822 IC_50_ value ranged from 1.5 to 12 μM for ALT-positive cell lines and from 1.3 to 8 μM for the ALT-negative cell lines with no significant difference between the two groups (Table 1).

### VE-822 is synergistic with gemcitabine in STS cell lines, increase DNA damage and prevents the checkpoint activation elicited by gemcitabine

**Figure 2:**
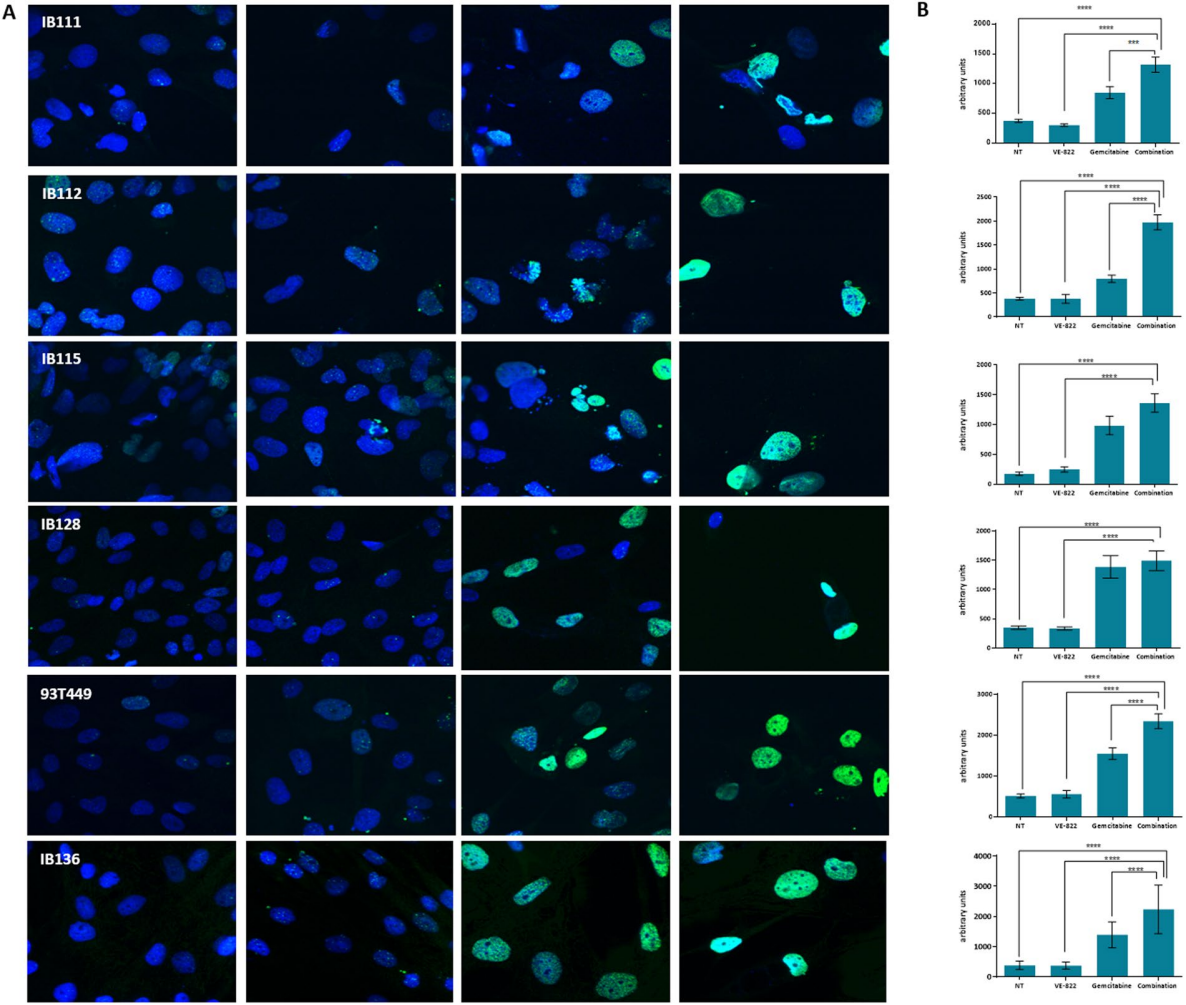
VE-822 augments DNA damage. **(A)** IB111, IB112, IB115, IB128, 93T449 and IB136 cells were immunostained with anti -γH2AX-specific antibodies before and after treatment with gemcitabine at 1/10 IC50 (0.28 nM, 0.11 nM, 0.19 nM, 0.33 nM, 0.82 nM and 0.37 nM respectively); VE-822 at 1/10 IC50 (0.47 µM, 0.58 µM, 0.15 µM, 0.3 µM, 0.51 µM and 0.8 µM respectively) and both drugs in combination. **(B)** Quantification of γ-H2AX marking has been realized by the integration of the total signal in IB111, IB112, IB115, IB136, 93T449, and IB136 cell lines. The experiments were performed in duplicate.

**Figure 3:**
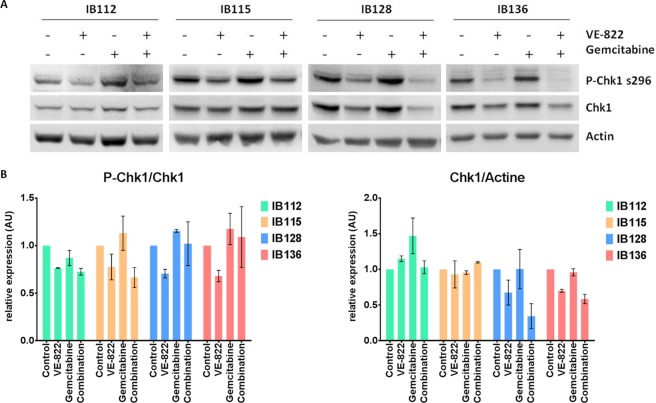
VE-822 inhibits the gemcitabine-induced checkpoint activation. **(A)** Western blot analysis of the phosphorylation of the CHK1 protein in IB112, IB115, IB128, and IB136 cells, which were either untreated or exposed to IC50 of Gemcitabine and/or IC50 of VE-822 for 24 h. **(B)** Quantification of Western blot analyses; the experiments were performed in duplicate.

The current clinical development of ATR inhibitors is based on combination regimens with additional therapies. We studied the effects of the combination of gemcitabine and VE-822 on the viability of STS cells. Eight STS cell lines were exposed during 72 hours to different combinations of both agents at a constant ratio of 1: 1 VE-822 and gemcitabine were mixed and diluted serially (usually 2 fold-serial dilutions with several concentrations above and below the IC50 for the two drugs). Combination Indices (CIs) were determined according to Chou *et al*.^8^. The results are described in Table 1. Interestingly, we observed an additive or synergistic effect in all the STS cell lines. We then assessed the level of DNA damage induced by the combination. By analyzing γ-H2AX expression, we found that the combination of VE-822 and gemcitabine induced significantly higher levels of DNA damage than either drug used as single agent alone (Fig. 2). We next investigated whether VE-822 could counteract the checkpoint activation elicited by gemcitabine. Importantly, in all the cell lines tested, VE-822 reduced the level of gemcitabine-induced CHK1 phosphorylation, the downstream ATR target (Fig. 3).

### VE-822 and gemcitabine combination induces apoptosis and cell cycle arrest in STS cell lines

We studied the effects of VE-822 and gemcitabine combination on apoptosis induction after 72 h of drug exposure as well as cell cycle effects after 48 h of treatment. We observed that the drug combination (picomolar amounts of Gemcitabine and micromolar amounts of VE-822) increased the rate of apoptosis in comparison with the drugs used as single agent in all the cell lines but one (IB128, Fig. 1). Furthermore, accumulation in S phase was also observed after treatment with the drug combination (Fig. 1) in all the cell lines except in IB136.

### VE-822 and gemcitabine combination reduces tumor growth in a patient-derived xenograft model of undifferentiated pleomorphic sarcoma

**Figure 4:**
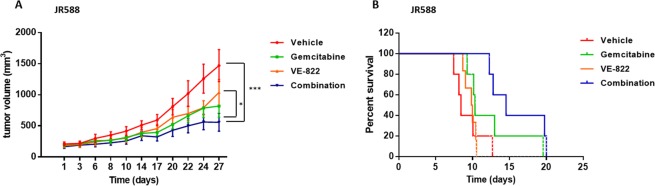
VE-822 is synergistic with gemcitabine in a patient-derived xenograft model (PDX) of undifferentiated pleomorphic sarcoma (UPS). **(A)** Effect of the combination of gemcitabine and VE-822 on tumor growth in the UPS PDX model JR588. **(B)** Kaplan-Meier survival curves for different mouse cohorts in the UPS PDX model JR588.

To further validate our *in vitro* results, we performed *in vivo* studies to test the antitumor effects of the VE-822 and gemcitabine combination. Xenografts were generated by subcutaneous implantation in ragγ2C−/− mice of one patient derived undifferentiated pleomorphic sarcoma. Animals were randomized in 4 groups and treated for 3 weeks. These groups included control (NaCl 0.9%), VE-822 (VE-822 alone; 60 mg/kg every day during 3 weeks), gemcitabine (gemcitabine alone; 30 mg/Kg IP, one time per week), and combination. After three weeks of treatment we observed a significant effect on progression free survival (evaluated as the time span from the treatment start and the doubling of the initial tumor volume), median time to doubling was 14.5 days for combination, 9.9 days for VE-822 (p = 0.0014) 10.3 days for gemcitabine, and 8.4 days for the vehicle (Fig. 4). No signs of toxicity were observed with the combination treatment.

## Discussion

Genome instability is a crucial hallmark of cancer. Physiologically, DNA damage response pathways maintain genome integrity by repairing DNA damage. Cancer cells are characterized by defects in DDR which results in increased mutational load, replication stress and genome instability. Chibon *et al*. have shown previously that high level of genome instability is associated with adverse outcome in patients with sarcomas^4^.

ATR is an essential DDR kinase activated in cancer cells with high level of replication stress. Therefore, several drugs targeting ATR are currently under clinical development. The first-in-class ATR inhibitor, VX-970 (Vertex Pharmaceutical; now M6620, Merck), has shown target modulation and promising preliminary signs of clinical activity in early-phase studies assessing its safety and efficacy as a single agent and in combination with chemotherapy^9^. Two other oral ATR inhibitors, AZD6738 (Astrazeneca), and BAY-1895344 (Bayer) are currently being tested in phase I or II trials as monotherapy or in combination with DDR agents, radiotherapy, chemotherapy or immunotherapeutics^10,11^. We report here the first study investigating the preclinical activity of ATR inhibition in a panel of pre-clinical models representing the most frequent histological subtypes of STS. Interestingly, we found that all the cell lines tested here displayed some degree of basal ATR activation as illustrated by the constitutive phosphorylation of CHK1 in sarcoma cells in the absence of treatment/damage induction. A recent study has shown that during unperturbed replication, some ATR and CHK1 molecules are activated to limit the activation of the replicative helicase in regions of ongoing DNA replication^12^. Further studies are needed to understand more in depth how is ATR and CHK1 signaling activated in unperturbed sarcoma cells. We found that all the cell lines tested displayed various degree of sensitivity to VE-822 with a median IC50 of 4.9 (range 1.3–12).

A recent study has suggested that ATR inhibitors are selectively active in cancer cells that employ the alternative lengthening telomere (ALT) pathway^7^. ALT is particularly common in bone and soft-tissue sarcomas^13^. Contrary to what was observed in osteosarcoma models, our results do not indicate a general hypersensitivity of ALT-positive STS cells when ATR is inhibited. Interestingly, there are in agreement with a recent study which did not identify any correlation between the ALT-or telomerase status of various cell lines and response to the ATR inhibitor VE-821^14^. In that study, the authors compared also the ATR inhibitor sensitivity in isogenic cell lines, in which ALT was active or suppressed and found absolutely no differences. Altogether, these findings do not support a synthetic lethality of ATR targeting in alternative lengthening of telomeres-dependent tumors and suggest that other pathways are involved in sensitivity to ATR inhibition. When the activity of ATR is inhibited, maintenance of genome integrity becomes dependent on functional TP53, with TP53 being essential for arresting cell cycle progression to permit repair^15^. ATR, therefore, represents a promising synthetically lethal target for TP53 deficiency^16,17^. Several studies have confirmed such synthetically lethal interaction by deleting ATR in TP53-deficient mice^17^ and by inhibiting its activity in tumor cell lines, which resulted in death induction of cells harboring TP53 defects^18–20^. TP53 pathway abrogation appears as a pivotal event in soft tissue sarcoma oncogenesis^21^. However, our results indicate that defective *TP53* as result of deletion or mutation or *MDM2* gene amplification do not confer greater sensitivity of STS cells to VE-822. This is in line with a recent study investigating the role of TP53 in sensitivity to four different ATR inhibitors in several models of osteosarcomas, breast, and colorectal cancers^22^. The authors were not able to find a correlation between *TP53* status and ATR inhibitor sensitivity even if gemcitabine sensitization was more pronounced in TP53-defective models. Altogether, these data suggest that TP53 is probably not a key determinant of the effect of ATR inhibition in tumor cells but only one contributor among other factors depending on the tumor type and the cellular context.

As even for the most sensitive STS lines, IC50 values were above 1 μM, we reasoned that achieving anti-tumor efficacy *in vivo* using VE-822, would be unlikely. Therefore, we sought to investigate the synergistic activity of VE-822 and gemcitabine when used in combination in STS models. In the present study we observed a synergistic or additive effect in all the cell lines tested. VE-822 strongly potentiated sub-IC_50_ levels of gemcitabine to induce S-phase arrest in the majority of the cell lines tested. Moreover, VE-822 synergized with gemcitabine to induce apoptosis in STS cells and does not only inhibit gemcitabine induced checkpoint activation, but also pre-existing CHK1 phosphorylation and/or CHK1 protein levels in general, while enhancing gemcitabine-induced DNA damage. We validated these *in vitro* results in the *in vivo* setting by using a patient-derived xenograft model of UPS, the most aggressive STS subtype^23^. As observed *in vitro*, the combination of VE-822 with gemcitabine significantly inhibited tumor growth in comparison with both drugs used as single agent.

We report here pre-clinical evidence that the combination of an ATRinh and chemotherapy is synergistic in soft-tissue sarcomas. Our results provide a rational for a clinical trial investigating the combination of gemcitabine and an ATR inhibitor in patients with advanced STS.

## Methods

All methods were performed in accordance with the relevant French and European Union guidelines and regulations.

### Cells and cell culture

All of the STS cell lines used in this study were derived from surgical specimens of patients with STS who underwent surgery at Institut Bergonié, Bordeaux, France and who gave written informed consent (Table 1) as previously described^24^. Each cell line was characterized by array comparative genomic hybridization for every 10 replicates to verify that its genomic profile was still representative of the originating tumor sample. Cells were grown in RPMI medium 1640 (Sigma Life Technologies, Saint Louis, MO) in the presence of 10% fetal calf serum (Dutscher, France) in flasks. Cells were maintained at 37 °C in a humidified atmosphere containing 5% CO_2_.

### Reagents

Gemcitabine was supplied by Institut Bergonié Pharmacy and VE-822, was purchased from Euromedex (Souffelweyersheim, France),

### Cell viability

Antiproliferative and cytotoxic effects of VE-822 and gemcitabine were first determined on 8 STS cell lines using Cytation 3 technology (Colmar, France) as previously described^24^. Briefly, cells were seeded in 384-well plates and were then exposed to VE-822 and/or gemcitabine for 72 h. Cells were then marked with propidium iodide (PI) and Syto 24 fluorochromes for 30 min. Quantitative fluorescence and cell imaging were performed with Cytation 3 at λ = 617 nm for PI and 521 for Syto 24.

### Cell cycle analysis

Cell cycle distribution was studied by examining DNA content using fluorescence-activated cell sorting and analyzed using Cell Quest Pro software (BD Biosciences, San Jose, CA, USA) as previously described^24^. 2 × 10^5^ cells were seeded in 6-well plates, and after 24 h, the cells were treated for 48 h with two different concentrations of VE-822 and/or gemcitabine, centrifuged at 1500 g for 5 min, and washed twice with PBS. The cells were then fixed with 70% ethanol at 4 °C overnight. Following ethanol removal, the cells were washed twice with PBS. Next, 300 µl of a PI and ribonuclease-containing solution were added to the cells and then analyzed by FACS. The data were analyzed with FlowJo v.7.6.3. software, and the results were expressed in terms of percentage of cells in a given phase of cycle.

### Apoptosis

For apoptosis assessment, 1.5 × 10^5^ cells were seeded in 6-well plates as previously described^24^. After 24 h, cells were treated with two doses of VE-822 and/or gemcitabine for 72 h and exposed to FITC-Annexin V and PI according to the manufacturer’s protocol (BD Biosciences, Erembodegem, Belgium). This allows us to distinguish Annexin V-positive cells in early apoptosis from Annexin V- and PI-positive cells in late apoptosis. Cells were analyzed by flow cytometry using FL1 for Annexin V and FL2 for PI. Flow cytometry (FACScan; BD Biosciences) data were analyzed with FlowJo v.7.6.3. software. Primary data are available on request.

### Western blot

Treated and control cells were harvested in 60 µL of radio-immunoprecipitation assay (RIPA) lysis buffer (Harlow and Lane, 2006). The lysate was centrifuged (13 000 rpm, 15 min, 4 °C), and the supernatant was stored at −80 °C. Equal amounts of total protein (30 µg) were electrophoresed on 12% sodium dodecyl sulfate (SDS) polyacrylamide gels and transferred onto polyvinylidene difluoride (PVDF) membranes for CHK1 expression analysis. The blots were probed overnight at 4 °C with an anti-phospho-CHK1 (S296, ab79758, 1/1000 Abcam), and an anti-CHK1 (Ab47574, 1/500, Abcam) primary antibody diluted in PBST (100 mM phosphate, 27 mM KCl, 1.37 M NaCl, pH 7.4 after 1X dilution; 0.2% Tween-20) with 5% BSA. The horseradish peroxidase-conjugated secondary antibody (Santa Cruz Biotechnology) was diluted 1:5000. Bound antibodies were visualized on Fusion Fx7 imaging system (Fisher Bioblock Scientific, Waltham, MA, USA) using the ImmobilonTM Western enhanced chemiluminescence detection kit (Millipore Corporation, Billerica, MA, USA). The resulting bands were analyzed and quantified using ImageJ® 1.49 g software (National Institutes of Health, Bethesda, MD, USA).

### Confocal microscopy

Cells were seeded on coverslips and treated with one concentration of VE-822, gemcitabine, or a combination of the two drugs for 72 h as previously described^24^. The slides were then washed twice with PBS, fixed in 4% formaldehyde, and incubated with anti-phosphoγH2ax monoclonal antibody (Cell Signaling, Leiden, Netherlands) overnight and then with goat anti-rabbit Alexa Fluor 488 antibody (Invitrogen, Paisley, United Kingdom). The slides were then counterstained using 4,6-diamidino-2-phenylindole (Hoechst).

### C-Circle assay

The alternative lengthening telomere status of the STS cell lines was established by using the C-Circle DNA ALT Assay (Capital Biosciences, Maryland, USA) as previously described^8^.

### PDX generation

IRB-approved informed consent to generate patient-derived murine xenografts was obtained from the relevant patients as previously described^24^. Animal care and procedures were approved by the Institutional Animal Care and Use Committee Office of the University of Bordeaux, France. Tumor specimens from three patients with UPS were cut into 3 × 3 × 3 mm pieces that were transferred to RPMI. Tumor pieces were inserted into incisions on the right flank of 5 nude mice.

### *In vivo* study

Four- to five-week-old female Ragγ2C−/− mice were used. Induction of tumor xenografts was performed by subcutaneous implantation of UPS tumor fragment (PDX) into the right flank of the mice. This study followed the French and European Union guidelines for animal experimentation (RD 1201/05, RD 53/2013 and 86/609/CEE, respectively). Mice were randomized into control and treatment groups (n = 6) two weeks after the tumor became measurable (15 days after injection: day 1 of treatment). Mice were randomized in 4 groups: vehicle (NaCl0.9%), VE-822 alone (60 mg/kg), gemcitabine alone (30 mg/kg), and both drugs VE-822 and gemcitabine were administered using 5%DMSO, 45% PEG 300 and NaCl0.9% as the vehicle respectively. The tumors were measured every 2–3 days with a caliper, and diameters were recorded. Tumor volumes were calculated using the formula: a^2^b/2, where a and b are the 2 largest diameters as previously described^24^. The mice were sacrificed by cervical dislocation after treatment arrest. Progression free survival curves were established based on two-fold tumor increase as event. All experimental manipulations with mice were performed under sterile conditions in a laminar flow hood.

### Statistical analysis

Data were analysed using the Student t-test for comparison of two means and ANOVA followed by the Turkey’s multiple comparison tests for more than two groups as previously described^24^; all the experiments were repeated in duplicate or triplicate. Data are represented as mean ± SD and significant differences are indicated as *p < 0.05, **p < 0.01 and ***p < 0.001.

Analysis of progression free survival was using LogRank test (Mantel-Cox test).

### Ethics approval and consent to participate

This study was approved by the IRB of Institut Bergonié, Bordeaux, France.
